# Integrative analysis of DNA methylation and gene expression reveals distinct hepatocellular carcinoma subtypes with therapeutic implications

**DOI:** 10.18632/aging.102923

**Published:** 2020-03-22

**Authors:** Xiaowen Huang, Chen Yang, Jilin Wang, Tiantian Sun, Hua Xiong

**Affiliations:** 1 State Key Laboratory of Oncogenes and Related Genes, Key Laboratory of Gastroenterology and Hepatology, Ministry of Health, Division of Gastroenterology and Hepatology, Renji Hospital, Shanghai Jiao Tong University School of Medicine, Shanghai Jiao Tong University, Shanghai Cancer Institute, Shanghai Institute of Digestive Disease, Shanghai, China; 2 State Key Laboratory of Oncogenes and Related Genes, Shanghai Cancer Institute, Renji Hospital, Shanghai Jiao Tong University School of Medicine, Shanghai, China

**Keywords:** hepatocellular carcinoma, DNA methylation-driven genes, classification, integrative analysis, gene expression

## Abstract

We aimed to develop an HCC classification model based on the integrated gene expression and methylation data of methylation-driven genes. Genome, methylome, transcriptome, proteomics and clinical data of 369 HCC patients from The Cancer Genome Atlas Network were retrieved and analyzed. Consensus clustering of the integrated gene expression and methylation data from methylation-driven genes identified 4 HCC subclasses with significant prognosis difference. HS1 was well differentiated with a favorable prognosis. HS2 had high serum α-fetoprotein level that was correlated with its poor outcome. High percentage of *CTNNB1* mutations corresponded with its activation in WNT signaling pathway. HS3 was well differentiated with low serum α-fetoprotein level and enriched in metabolism signatures, but was barely involved in immune signatures. HS3 also had high percentage of *CTNNB1* mutations and therefore enriched in WNT activation signature. HS4 was poorly differentiated with the worst prognosis and enriched in immune-related signatures, but was barely involved in metabolism signatures. Subsequently, a prediction model was developed. The prediction model had high sensitivity and specificity in distributing potential HCC samples into groups identical with the training cohort. In conclusion, this work sheds light on HCC patient prognostication and prediction of response to targeted therapy.

## INTRODUCTION

Hepatocellular carcinoma (HCC) is the sixth most common cancer and the third leading cause of cancer-related death worldwide [1]. It is estimated that by 2020 the number of HCC cases will reach 78,000 in Europe and 27,000 in the United States [1]. A better understanding of the underlying mechanisms of HCC diversity will increase the chances for effective treatment and improvement in survival rate.

Genome-wide analyses of mRNA expression profiles have contributed to developing HCC targeted therapies over the past two decades. Boyault et al. performed transcriptome analyses on 57 HCCs and 3 hepatocellular adenomas. Six robust subgroups of HCC (G1-G6) associated with clinical and genetic characteristics were identified [2]. Hoshida et al. classified a total of 603 patients into 3 robust HCC subclasses (S1, S2, and S3) based on gene expression profiles. Each subclass was correlated with clinical parameters such as tumor size and extent of cellular differentiation [3]. Chiang et al. divided 91 HCC samples into 5 subclasses based on gene expression profiles [4]. Lee et al. analyzed global gene expression patterns of 91 HCCs. The samples were classified into two distinctive subclasses that were highly associated with patient survival [5]. The existing classifications are mainly based on gene expression profiles, and few of them are based on DNA methylation profiles. However, HCC is a complex disease arising from accumulation of both genetic and epigenetic alterations [6]. Transcriptome data alone is insufficient for revealing the heterogeneity of HCC. It has been demonstrated that classification of HCC with DNA methylation data is clinically significant [7].

As one of the core elements in epigenetic modifications, DNA methylation participates in a diverse range of cellular and biological processes such as cell differentiation, aging, tissue-specific gene expression, genome stability and genomic imprinting [8]. In addition to the implication during normal development, DNA methylation involves in pathologies such as carcinogenesis [9]. Hypermethylation of CpG islands in promoter sequences can cause epigenetic inactivation of tumor suppressor genes followed by mRNA transcript repression [9]. Unlike DNA aberrations, epigenetic changes are reversible, which makes them potential therapeutic targets [9].

Aberrant methylation of several tumor suppressor genes and tumor-related genes such as *RASSF1A*, *hMLH1* and *SOCS1* is constantly identified in HCC [10]. *TMS1* is a proapoptotic gene with promoter methylation observed in 80% HCC patients [11]. Aberrant methylation of *SEMA3B* is reported in 80% HCCs [11]. *SEMA3B* induces apoptosis and is detected in lung cancers and gliomas [11]. A number of studies on these DNA methylation-driven genes have already been published [12, 13].

To obtain a better understanding of HCC heterogeneity, we established an HCC classification based on integrated gene expression and methylation data of methylation-driven genes (MDGs). Consensus clustering identified 4 HCC subclasses significantly associated with prognosis value. The 4 subclasses showed distinct clinical features and enrichment in different signatures. Somatic mutations and copy number mutations data were analyzed and visualized. Besides, HCC patients were clustered into distinct CpG island methylator phenotype (CIMP) based on the methylation level of 674 most variable CpGs. The accuracy of the transcriptome-based prediction model constructed by machine learning algorithms was favorable.

## RESULTS

### Identification of 4 HCC subclasses

**Figure 1:**
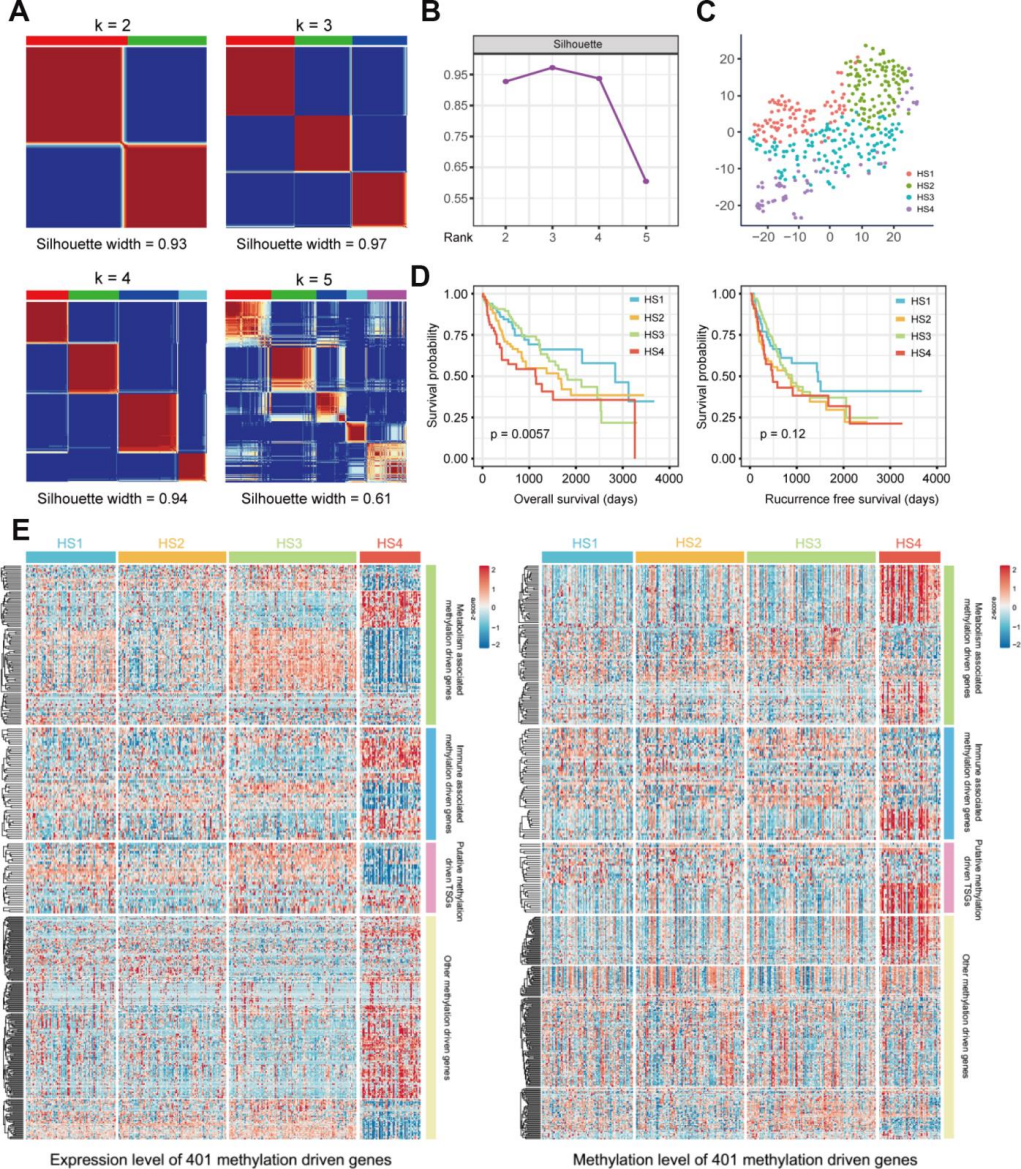
**Identification of HCC subclasses based on integrated transcriptome and methylation data of MDGs.** (**A**) Consensus matrix for k = 2 to k = 5. (**B**) Silhouette values under corresponding k values. (**C**) T-SNE analysis of mRNA expression data from tumor samples included in the cluster analysis (**D**) OS and RFS of 4 HCC subclasses. Statistical significance of differences was determined by Log-rank test. (**E**) Heatmaps show the expression and methylation level of 401 MDGs in HCC subclasses. 401 MDGs were divided into 4 groups, including metabolism associated MDGs, immune associated MDGs, putative methylation driven TSGs and other MDGs. HCC: hepatocellular carcinoma; MDG: methylation driven gene; t-SNE: t-distributed stochastic neighbor embedding; OS: overall survival; RFS: recurrence free survival; TSG: tumor suppressor genes.

Messenger RNA expression data and methylation data were integrated under the same sample with the *MethylMix* R package [14] to identify MDGs. 401 MDGs with |logFC| > 0, P < 0.05 and |Cor| > 0.3 were reserved for subsequent analyses (Supplementary Table 1). Then, 369 HCC patients were clustered based on the integrated mRNA expression and methylation data of 401 MDGs by “ExecuteCNMF” function in *CancerSubtypes* package [15]. Optimal number of clusters was determined according to comprehensive consideration of Silhouette width value and clinical significance (Figure 1A, 1B and Supplementary Figure 1). When the samples were classified into 2, 3 and 4 subtypes, average silhouette widths were 0.93, 0.97 and 0.94, respectively. If Silhouette width is close to 1, it means the samples are well classified. Silhouette widths for 2, 3 and 4 clusters were all close to 1. Besides, when the samples were classified into 3 groups, no significance in survival was identified (p=0.0692). We considered it more appropriate to divide the samples into 4 subclasses to provide more information for diagnosis based on their different molecular features. The 4 HCC subclasses identified were named HCC Subclass 1 (HS1), HCC Subclass 2 (HS2), HCC Subclass 3 (HS3) and HCC Subclass 4 (HS4). To validate subclasses’ assignments, we performed t-distributed stochastic neighbor embedding (t-SNE) to decrease the dimension of features and found that subtype designations were largely concordant with two-dimensional t-SNE distribution patterns (Figure 1C).

Survival analysis was conducted, and significant prognostic difference was observed when using overall survival (OS) as an endpoint (log-rank test *P* = 0.0057, Figure 1D). A longer median survival time (MST) was detected for HS1 (MST=2839 days, 95% CI: 1749-3929 days) compared with HS2 (MST= 1622 days, 95% CI: 929-2315 days, *P* = 0.0609), HS3 (MST=1818 days, 95% CI: 1213-2423 days, *P* = 0.5308) and HS4 (MST= 1135 days, 95% CI: 450-1820 days, *P* = 0.0034). However, when using recurrence free survival (RFS) as an endpoint, there was no significant prognostic difference among HCC classifications (Figure 1D and Supplementary Table 2).

The characteristics of 401 MDGs were then investigated. Metabolism and immune relevant gene lists were obtained from previous studies [16, 17]. Through intersecting these gene lists with 401 MDGs, we identified metabolism and immune associated MDGs (100 MDGs for metabolism and 51 for immunity). Besides, considering that DNA methylation alterations in tumor suppressor genes (TSGs) were involved in carcinogenesis, we intersected 401 MDGs with putative TSGs to obtain putative methylation driven TSGs. The expression and methylation levels of these MDGs were both visualized in Figure 1E and detailed information was listed in Supplementary Table 3.

### Correlation of the HCC subclasses with clinical characteristics and classical classification

**Figure 2:**
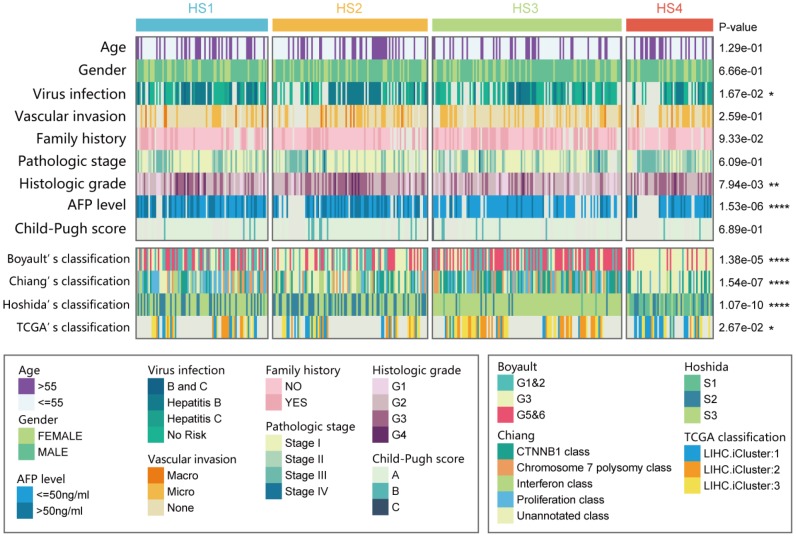
**Correlation of our classification (HS1, HS2, HS3 and HS4) with distinct clinical characteristics and previously published HCC subclasses.** Prediction of previously published HCC classifications was performed with NTP analyses. Statistical significance of differences was determined by Chi-square test (ns represents no significance, * represents P < 0.05, ** represent P < 0.01, *** represent P < 0.001, **** represent P < 0.0001). HCC: hepatocellular carcinoma; NTP: Nearest Template Prediction.

The relationships between HCC classifications and clinical characteristics were then investigated (Figure 2 and Supplementary Table 4). Results revealed that HS2 was associated with histologic grade G3/G4 (46/99 vs 82/259, *P* = 0.0089) and high serum α-fetoprotein (AFP) level (37/75 vs 58/201, *P* = 0.0014). HS3 was associated with lower proportion of virus infection (44/87 vs 58/166, P = 0.0160), histologic grade G1/G2 (93/120 vs 137/238, P = 0.0002), and low serum AFP level (77/90 vs 105/186 in the rest, P < 0.0001).

Then, our classification was also compared with previously reported HCC molecular subclasses, including Boyault’s classification [2] (G1 to G6), Chiang’s classification [4] (5 classes), Hoshida’s classification [3] (S1, S2, and S3), and The Cancer Genome Atlas (TCGA) classification [18] (iCluster1, iCluster2, and iCluster3). Results suggested that HS1 was significantly associated with Chiang's Proliferation class (31/85 vs 52/278 in the rest, *P* = 0.0006). HS2 was significantly associated with Hoshida’s S2 (47/100 vs 53/263 in the rest, *P* < 0.0001). HS3 was significantly associated with Boyault’s G5/G6 (66/122 vs 65/241 in the rest, *P* < 0.0001), Chiang's *CTNNB1* class (45/122 vs 44/241 in the rest, *P* = 0.0001), and Hoshida’s S3 (111/122 vs 114/241 in the rest, *P* < 0.0001). HS4 was significantly associated with Boyault’s G3 (45/56 vs 97/307 in the rest, *P* < 0.0001), Hoshida’s S1 (28/56 vs 10/307 in the rest, *P* < 0.0001), and TCGA iCluster1 (20/33 vs 41/145 in the rest, *P* = 0.0004).

### Correlation between HCC subclasses and CIMP

Considering that MDGs based classification may result in different methylation status among subclasses, we then explored the methylation characteristics of 4 HCC subclasses. First, according to previously mentioned approach to find CIMP in HCC [7], we clustered samples into distinct groups using K-means method based on the methylation level of 674 most variable CpGs. Among these groups, C2 was defined as non-CIMP group with the lowest methylation level of 674 CpGs. C7 was defined as CIMP-H group with the highest methylation level of 674 CpGs. The remaining groups with moderate methylation level of 674 CpGs were defined as CIMP-L group (Supplementary Figure 2A). Although no significant prognostic difference was observed among groups, CIMP-H (C7) group still showed a trend towards poorer prognosis (Supplementary Figure 2B and 2C). The relationship between our classification and CIMP was visualized in Supplementary Figure 2D, and results of statistical analysis revealed that samples in non-CIMP were more enriched in HS3 and HS4 than HS1 and HS2 (63/180 vs 44/189, *P* = 0.0131).

### Correlation of HCC subclasses with metabolism and immune associated signatures

**Figure 3:**
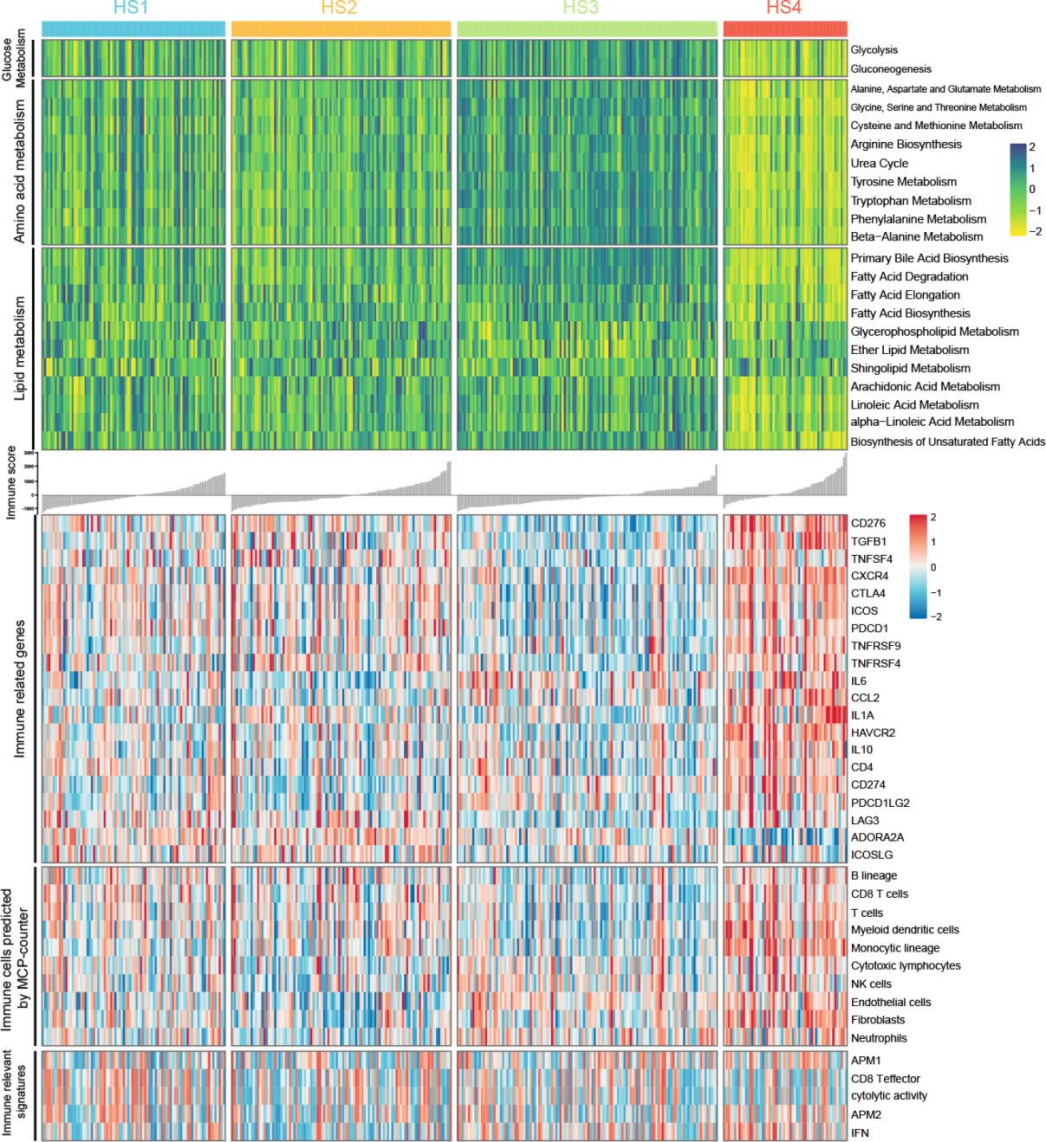
**Heatmaps show difference in metabolism signatures (glucose metabolism, amino acid metabolism, and lipid metabolism), immune related genes expression, immune-associated signatures and other signatures, immune and stromal cell populations predicted by MCP-counter among 4 HCC subclasses (see detailed information in Supplementary Figure 2).**

The outcome that 100 of the 401 MDGs were involved in metabolism and 51 were involved in immunity drove us to investigate the characteristics of metabolism and immunity in HCC subclasses (Figure 3). First, metabolism and immune associated processes were quantified using Gene Set Variation Analysis (GSVA) and microenvironment cell populations-counter (MCP-counter) methods. Then, statistical analyses were conducted, and results suggested that metabolic and immune processes in distinct classifications differed greatly (detailed statistical analyses were shown in Supplementary Figure 3A). Particularly, HS3 had higher signature scores for metabolism than other subclasses, except several lipid metabolic processes, including glycerophospholipid metabolism, ether lipid metabolism, shingolipid metabolism, arachidonic acid metabolism, and alpha−linoleic acid metabolism. HS4 exhibited lower enrichment in these metabolic processes than other subclasses. HS1 and HS2 had moderate signature scores, and there was also no significant difference between HS1 and HS2.

For immune associated processes, we first investigated the association between subclasses and the expression of 20 potentially targetable immune related genes, and results indicated that HS4 exhibited higher expression for multiple immune related genes (*CD276, TGFB1, CXCR4, CTLA4, ICOS, TNFRSF9, CCL2, IL1A, HAVCR2, IL10, CD274*, and *PDCD1LG2*) and lower expression for *ADORA2A* than other subclasses (Supplementary Figure 3B). HS3 exhibited lower expression for *CD276, TGFB1, CTLA4, ICOS, PDCD1, TNFRSF4, CD274*, and *LAG3* than other subclasses. No significant difference for immune related gene expression was detected between HS1 and HS2. We then explored immune infiltration of 4 subclasses. The abundance of 10 immune and stromal related cell types was calculated using MCP-counter algorithm. Significant difference was observed between HS4 and other 3 subclasses, with higher abundance of 4 cell populations (T cells, myeloid dendritic cells, monocytic lineage, and Fibroblasts) for HS4 compared with other 3 subclasses. In addition, HS3 exhibited lower enrichment of B lineage, CD8 T cells, T cells, and myeloid dendritic cells. There was no significant difference of cell abundance in most cell populations between HS1 and HS2 (Supplementary Figure 3C). For immune associated signatures, HS4 exhibited higher enrichment for interferon (IFN) signature than HS1 and HS2 (Supplementary Figure 3D).

### The difference of other critical signatures among HCC subclasses

**Figure 4:**
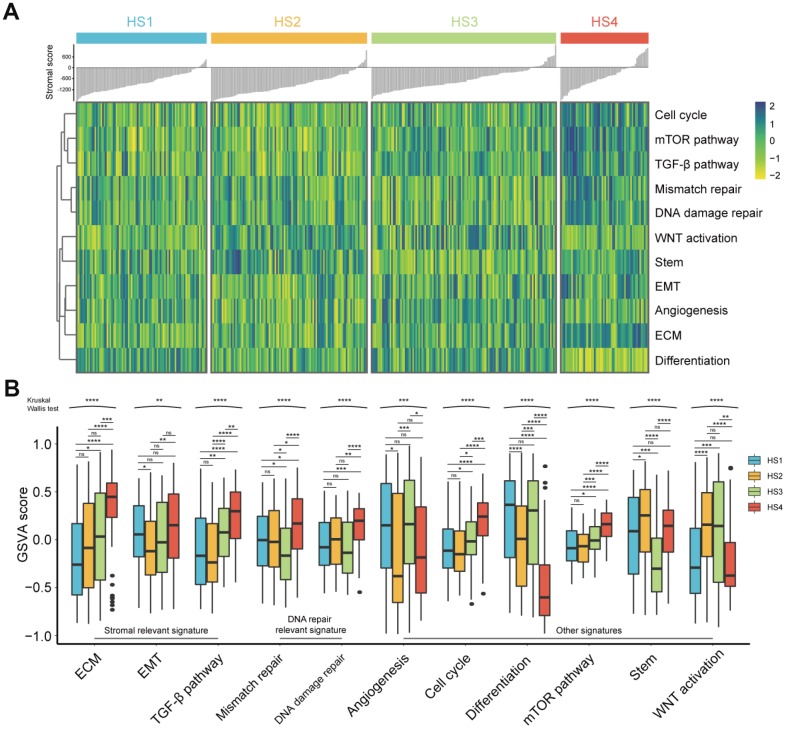
**Difference of progression-relevant signatures among HCC subclasses.** (**A**) Heatmap of progression-relevant signatures in 4 HCC subclasses. (**B**) Box plots (from 25^th^ percentile to the 75^th^ percentile with a line at the median) show the abundance of progression-associated signatures. Statistical significance of overall differences was determined by Kruskal Wallis test (ns represents no significance, * represents P < 0.05, ** represent P < 0.01, *** represent P < 0.001, **** represent P < 0.0001).

The associations between our HCC classification and several critical signatures involved in oncogenesis and progression of HCC were also investigated, including extracellular matrix (ECM) signature, epithelial mesenchymal transition (EMT) signature, TGF-β signature, mismatch repair signature, DNA damage repair signature, angiogenesis signature, cell cycle signature, differentiation signature, mTOR pathway signature, stem signature, and WNT activation signature (Figure 4A and 4B). Results showed that HS4 demonstrated a higher enrichment of stromal relevant signature (ECM signature and TGF-β signature), DNA repair relevant signature, cell cycle signature, mTOR signature and lower enrichment of differentiation signature compared with other 3 subclasses. HS3 exhibited lower enrichment of stem signature than other subclasses, and higher enrichment of differentiation signature than HS2. In addition, no significant difference of WNT activation signature was observed between HS2 and HS3, and both of them showed a higher enrichment of WNT activation signature than HS1 and HS4. HS1 and HS2 showed no significant difference in enrichment of ECM signature, TGF-β signature, DNA repair relevant signature, cell cycle signature, and mTOR signature. HS2 showed higher enrichment of stem signature compared with HS4. HS1 showed higher level of angiogenesis signature and differentiation signature compared with HS3.

**Figure 5:**
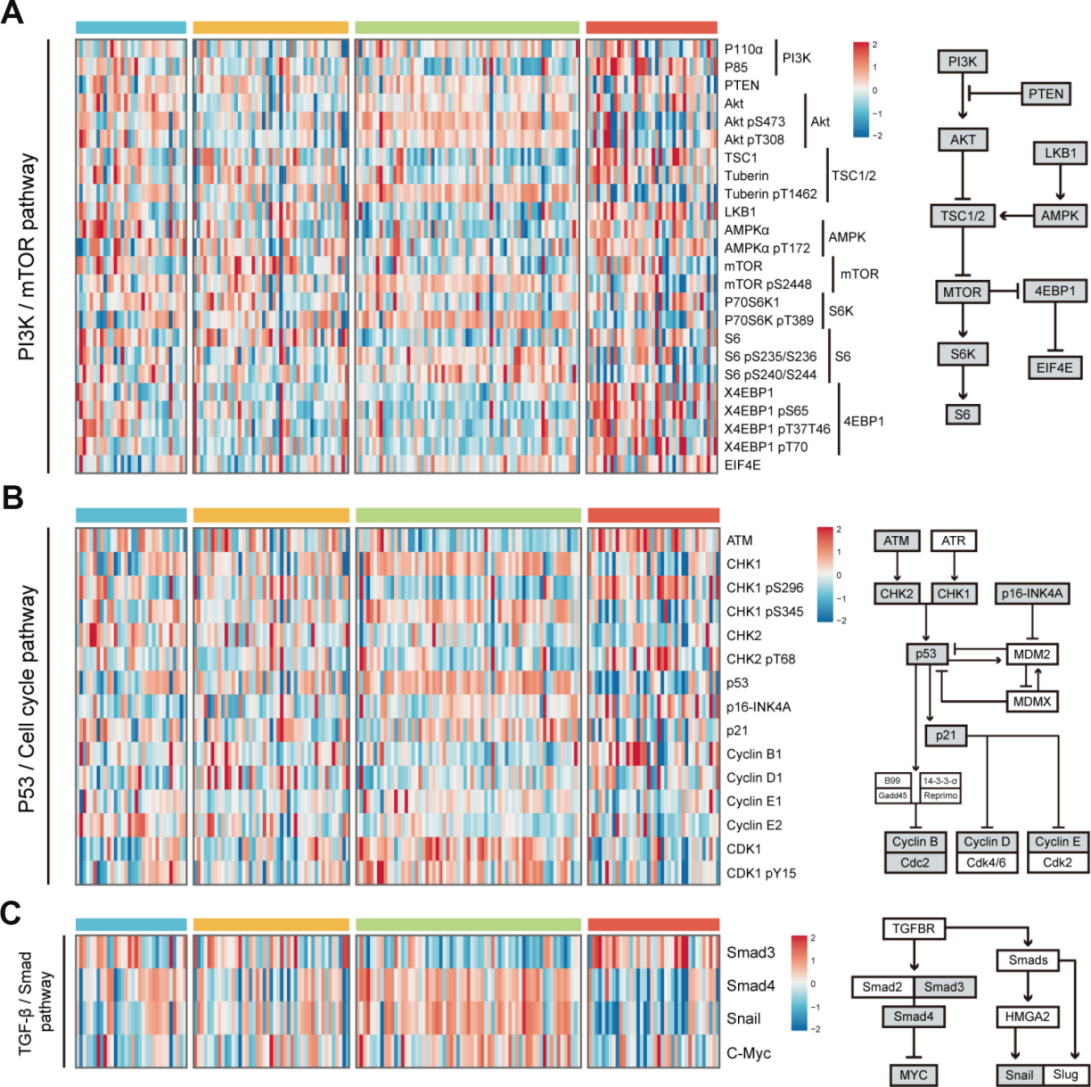
**Difference of protein expression levels in PI3K/mTOR pathway, P53/Cell cycle pathway, and TGF-β/Smad pathway among 4 HCC subclasses.** (**A**) Heatmap shows the expression level of 24 proteins in PI3K/mTOR pathway. The right half of the figure shows the basic components of PI3K/mTOR pathway. (**B**) Heatmap shows the expression level of 15 proteins in P53/Cell cycle pathway. The right half of the figure shows the basic components of P53/Cell cycle pathway. (**C**) Heatmap shows the expression level of proteins in TGF-β/Smad pathway. The right half of the figure shows the basic components of TGF-β/Smad pathway (see detailed information in Supplementary Figure 3). Data is available for proteins inside grey boxes. HCC: hepatocellular carcinoma.

Considering the limited evidence provided by transcriptome data, we further analyzed proteomic data to validate the conclusion. Reverse Phase Protein Array (RPPA) based proteomic data was download from The Cancer Proteome Atlas (TCPA) database. All proteins were annotated according to their corresponding genes. Because of the limited proteins detected by protein array, we only chose to investigate the difference of protein levels in PI3K/mTOR pathway, p53/Cell cycle pathway and TGF-β/Smad pathway among 4 HCC subclasses. In PI3K/mTOR pathway, HS4 exhibited higher expression of S6_pS240/S244, X4EBP1 and X4EBP1_pT70 than other 3 groups. HS3 had higher expression of AKT_pS473, Tuberin_pT1462 and P70S6K_pT389 than other groups (Figure 5A and Supplementary Figure 4A). In P53/Cell cycle pathway, HS4 had higher expression of ATM and CHK1_pS296, while HS3 had higher expression of CHK1, CHK1_pS345, P53, CDK1 and CDK1_pY15 (Figure 5B and Supplementary Figure 4B). In TGF-β/Smad pathway, HS3 had lower expression of Smad3 and higher expression of Snail than other 3 groups (Figure 5C and Supplementary Figure 4C). Significance was detected between HS1 and HS2 for the expression of AKT and AKT_pT308.

### Mutations and copy number alterations associated with HCC subclasses

To investigate differences in mutations and copy number alterations among HCC subclasses, we analyzed the somatic mutation and copy number data. The mutation status of genes in p53/Cell cycle pathway, Wnt/beta-catenin pathway, hepatic differentiation, and DNA methylation was visualized in Supplementary Figure 5A. Results of statistical analysis revealed that HS1 was associated with a low percentage of alterations in *CTNNB1* (13/84 vs 70/265 in the rest, *P* = 0.0402) and a high percentage of alterations in *AXIN1* (11/84 vs 17/265 in the rest, *P* = 0.0495). HS2 was associated with a high percentage of alterations in *AXIN1* (13/99 vs 15/250 in the rest, *P* = 0.0271). HS3 was associated with a low percentage of alterations in *TP53* (19/112 vs 81/237 in the rest, *P* = 0.0009), *MUC16* (10/112 vs 44/237 in the rest, *P* = 0.0201) and *AXIN1* (2/112 vs 26/237 in the rest, *P* = 0.0032), and a high percentage of alterations in *CTNNB1* (35/112 vs 48/237 in the rest, *P* = 0.0243). HS4 was associated with a low percentage of alterations in *CTNNB1* (5/54 vs 77/295 in the rest, *P* = 0.0174). Detailed results of the above statistical analyses were shown in Supplementary Table 5. Subsequently, mutation signatures in subclasses were investigated. First, we explored the proportion of 6 single-nucleotide substitutions (C>A/G>T, C>G/G>C, C>T/G>A, T>A/A>T, T>C/A>G, and T>G/A>C) in each HCC subclass (Supplementary Figure 5B). Then we computed sample-wise signature profiles, and filtered out mutation signatures with no prognostic significance (*P* > 0.15 in Cox regression). 4 mutation signatures (Signature 4, 18, 22, and 24) were remained after filtration (Supplementary Table 6), and signature weight was transformed into mutation number for comparison among groups. Significant difference of Signature 24 among 4 subclasses was observed, with more mutations of Signature 24 in HS4 than in HS3. (Supplementary Figure 5C and 5D).

Aside from point mutations and short insertions/deletions, we also analyzed DNA copy number alterations across distinct classifications based on segmentation data obtained from TCGA by using GISTIC2. Genome-wide focal amplification (red) and deletion (blue) peaks identified in different subclasses were presented in Supplementary Figure 6A. The number of specific amplification regions for HS1, 2, 3 and 4 were 18, 6, 37 and 8, respectively. The number of specific deletion regions for HS1, 2, 3 and 4 were 15, 7, 13 and 7, respectively. The common amplification regions of 4 subclasses were 5p15.33, 6q12, 11q13.3 and 19p13.12, while common deletion region was 9p21.3 (Supplementary Figure 6B and Supplementary Table 7).

### Class prediction of HCC patients based on transcriptome data

**Figure 6:**
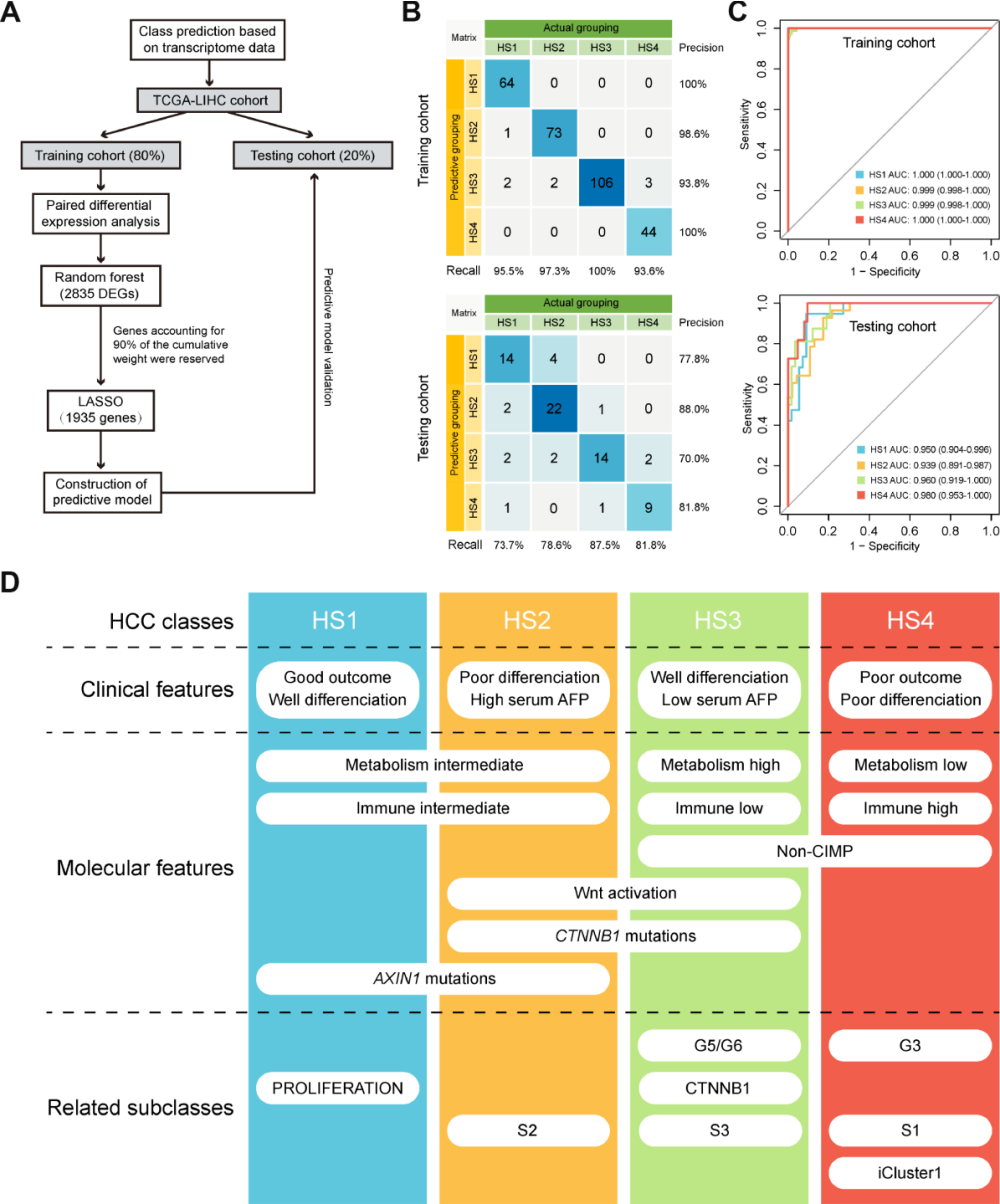
**Class prediction of HCC patients.** (**A**) Flow chart shows the process of prediction model construction. (**B**) Confusion matrix evaluations of prediction model within the training cohort and testing cohort. A perfect prediction model (100% accuracy) have 0 counts for all non-diagonal entries (that is, no misclassified samples). (**C**) ROC curves in training and testing cohort depict trade-offs between true and false positive rates as classification stringency varies. AUC values close to 1 indicate that a high true positive rate was achieved with low false positive rate, while AUC values close to 0.5 indicate random performance. (**D**) Overview of the characteristics of 4 HCC subclasses. HCC: hepatocellular carcinoma; ROC: Receiver operating characteristic; AUC: Area under the curve.

We labeled each sample with its assigned cluster according to the HCC classification we established. A classification model was developed to investigate whether potential HCC samples can be distributed into groups identical with the training cohort based on transcriptome data of 2835 differentially expressed genes (DEGs) (Supplementary Table 8). The workflow was shown in Figure 6A. A transcriptome-based prediction model was constructed by random forest (RF) and Least Absolute Shrinkage and Selector Operation (LASSO) algorithm. The accuracy of the model in training cohort and testing cohort were 97.3% and 79.7%, respectively (Figure 6B). Then, we performed receiver operating characteristic (ROC) curves that can illustrate the relationship between TPR (sensitivity) and FPR (1-specificity) for each class. Area under the curve (AUC) close to 1 indicates that the classifier is predicting with maximum TP and minimum FP. Results of AUC for HS1, 2, 3 and 4 in training cohort were 1.000, 0.999, 0.999 and 1.000, respectively. In the testing cohort, AUC for HS1, 2, 3 and 4 were 0.950, 0.939, 0.960 and 0.980, respectively (Figure 6C).

## DISCUSSION

This integrative analysis based on DNA methylation and gene expression profiles of MDGs in HCC revealed 4 subclasses with distinct features (Figure 6D). HS1 was well differentiated with the best prognosis and high percentage of *AXIN1* mutations. HS2 had high serum AFP level that was correlated with its poor outcome. High percentage of *CTNNB1* mutations corresponded with HS2’s activation in WNT signaling pathway. HS3 was well differentiated with low serum AFP level and enriched in metabolism signatures, but was barely involved in immune signatures. HS3 also had high percentage of *CTNNB1* mutations and enriched in WNT activation signature. HS4 was poorly differentiated with the worst prognosis and enriched in immune-related signatures, but was barely involved in metabolism signatures. HS3 and HS4 both enriched in non-CIMP. Machine learning algorithms were applied to building a prediction model, and results showed that the model had high sensitivity and specificity in distributing potential HCC samples into distinct classifications.

The best prognosis value of HS1 was associated with high GSVA score in differentiation and lower score in WNT activation signature. The poor prognosis of HS2 was associated with higher enrichment in stemness and WNT signaling pathway activation. WNT signaling activation is mainly due to mutations in *CTNNB1*, a β-catenin gene [19]. The frequent mutations of *CTNNB1* in HS2 corresponded with its activation in WNT signaling. HS3 patients also had high percentage of alterations in *CTNNB1*. HS3 presented lower score in stemness and higher score in differentiation. On the contrary, HS4 had higher score in ECM, TGF- β pathway, mismatch repair, DNA damage repair, cell cycle, mTOR pathway and lower score in differentiation. The worst prognosis of HS4 was correlated with its involvement in the above mentioning carcinogenesis signatures.

HS2 and HS3 patients may be beneficial from therapeutic approaches that aim to target the Wnt–β-catenin pathway. For example, a small peptide called CGX1321 can inhibit Wnt lipid modifications. It is tested by a phase I trial in patients with advanced solid tumors, including HCC (https://clinicaltrials.gov/ct2/show/NCT02675946). Another phase I trial tests the efficacy of OMP-54F28, a fusion protein targeting Wnt ligands (https://clinicaltrials. gov/ct2/show/NCT02069145). DKN-01 inhibits non-canonical β-catenin pathway and is currently being investigated in a phase I trial in combination with gemcitabine and cisplatin in various cancers including HCC (https://clinicaltrials.gov/ct2/show/NCT02375880). The efficacy of these therapies for HS2 and HS3 patients requires further investigation. HS4 patients may be beneficial from therapies targeting the mTOR pathway. It has been reported that Everolimus is an mTOR inhibitor that can prevent tumor progression and improve survival in preclinical HCC models. A phase III study tested the efficacy of Everolimus in patients with advanced HCC after failure of sorafenib [20].

HS3 exhibited higher expression level of Akt involving in PI3K/mTOR pathway, indicating that HS3 patients may be beneficial from Akt inhibitors. As the key component of PI3K signaling pathway, Akt is considered to be an attractive target for cancer therapy [21]. Multiple Akt inhibitors such as ATP-competitive inhibitors (GSK690693, GDC0068, and AZD5363) and allosteric inhibitors (MK-2206) have been investigated in clinical trials against tumors. The results are promising [22]. HS4 had higher expression of ATM, a core component of the DNA repair system [23]. Targeting ATM may be a promising strategy for cancer treatment [23]. Currently, ATM inhibitors such as AZD0156 and AZD1390 are under investigation in phase I clinical trials [23]. HS4 patients may be beneficial from ATM inhibitors. HS3 had higher expression of CDK1, indicating HS3 patients may be beneficial from inhibitors targeting CDK1. BEY1107, an anti-cancer agent that selectively acts on CDK1, is in phase I/II clinical trial [24].

It appears that human cancer mutations and cancer genes constantly affect metabolism processes including aerobic glycolysis, glutaminolysis and one-carbon metabolism that produce amino acids, nucleotides, fatty acids and other substances for cell growth and proliferation [25]. Metabolic therapies targeting certain metabolism process provide alternatives for chemoresistant patients. For example, it has been reported that metformin can prevent liver carcinogenesis [26] and treatment with metformin is associated with favorable prognosis in patients with HCC [27]. Determining the responders of metabolic therapies has proven to be challenging [28]. This study provided insights into predicting potential responders towards metabolic therapies. HS3 enriched in metabolism signatures including glucose metabolism and amino acid metabolism, indicating that HS3 patients may be beneficial from metabolic therapies like metformin. On the other hand, HS4 patients presented low enrichment in metabolic processes, suggesting that they may be non-responders towards metabolic therapies. These assumptions require further experimental validation.

In the last decades, immunotherapy has been investigated and applied in multiple tumors including HCC. Immune checkpoints play an essential role in maintaining tolerance and preventing T cell over-activation [29]. Immune checkpoint expression can lead to T cell exhaustion and immune tolerance [29]. PD-1 is expressed by activated T cells, B cells, NK cells and myeloid cells [29]. In physical conditions, when PD-L1 is expressed on antigen presenting cells (APC), the interaction between PD-L1 and PD-1 will maintain self-tolerance and prevent the activation of T cells [30]. However, tumor cells can also express PD-L1 thus inducing immune tolerance [31]. The multiplicity of infiltrating PD1^+^ CD8^+^ cells and the expression of PD-L1 in HCC cells have been proven to be associated with worse prognosis [32]. Other checkpoint molecules including CTLA4, TIM3 and LAG3 are also implicated in the suppression of immune response against HCC [29]. Immune checkpoint inhibitors (ICIs) can unleash cytotoxic T cells against tumors to strengthen immune response thus showing anti-tumor efficacy [33]. ICIs including CTLA-4 and PD-1/PD-L1 inhibitors have been investigated in clinical trials of HCC. Nivolumab is a PD-1 immune checkpoint inhibitor. Promising results regarding its efficiency have been achieved in a clinical trial on advanced HCC patients [34]. Pembrolizumab is another PD-1 immune checkpoint inhibitor which has been proven to be effective for advanced HCC patients who was previously treated with sorafenib [35]. In this study, HS4 showed higher expression for most of the immune checkpoint genes, while HS3 exhibited lower expression for *CD276, TGFB1, CTLA4, ICOS, PDCD1, TNFRSF4, CD274,* and *LAG3* than other subclasses. Results indicated that HS4 patients may be responders towards ICIs while HS3 patients were less likely to respond to ICIs. In addition, HS4 also exhibited higher enrichment for IFN signature than HS1 and HS2. IFNγ is one of the cytokines that can induce PD-1 expression in T cells [29], which may be associated with the highest expression of immune checkpoint genes in HS4. The worst prognosis of HS4 was associated with its high expression of immune checkpoint molecules.

CIMP is a phenomenon of simultaneous methylation in multiple genes [7]. Although the fraction of CIMP is smaller in HCC compared with other cancer types, the CIMP group still requires special attention because of its poor prognosis [7]. Based on the methylation level of 674 most variable CpGs, HCC patients were clustered into 7 groups, which was consistent with a previous study [7]. Although no significant outcome was identified, consistent with previous results [7], the overall survival time of CIMP patients was statistically shorter than that of non-CIMP patients. Several drugs that modify DNA methylation by targeting DNA methyltransferases have been investigated. For example, it has been reported that Zebularine (1-(β-(D)-ribofuranosyl)-1,2-dihydropyrimidin-2-one) inhibits DNA methylation and induces apoptosis in HepG2 cell line [36]. Another study reported that Zebularine inhibits tumor growth in xenograft models. Genes involved in apoptosis, cell cycle, and tumor suppression were demethylated in liver cancer cell lines [37].

In conclusion, this classification based on integration of DNA methylation and transcriptome profiles revealed distinct characteristics of HCC subtypes, which provided novel clinical insights into predicting both the prognosis of HCC and prospective therapies. Future research will accelerate the clinical validation of HCC classification and will promote precision diagnostics as well as therapeutics for HCC patients.

## MATERIALS AND METHODS

### Data preparation

Multiplatform genomics data, including mRNA expression data (raw counts), gene somatic mutation data (MAF files), DNA copy data (segment file) (March 27, 2019), DNA methylation array data (July 27, 2019), RPPA data and corresponding clinical information (August 19, 2019) of TCGA-LIHC cohort were retrieved from TCGA database (http://cancergenome.nih.gov/).

Transcripts per kilobase million (TPM) values were calculated based on raw counts. DNA methylation array data was generated from the Illumina Infinium HumanMethylation450 BeadChip array. Methylation level of each probe was represented by β value (ranging from 0 to 1). Probes containing ‘NA’ marked data points or located on sex chromosomes were removed. Then, probes residing in gene promoter regions including the upstream 2.5 kb from TSS, 5’UTR and first-exon regions were mapped to their corresponding genes. Methylation level of a certain gene was determined as the average methylation level of corresponding probes residing in promoter regions.

### Identification of methylation driven genes-associated classification

First, MDGs were identified based on mRNA expression data from tumor samples and methylation data from tumor and normal samples by using *MethylMix* package in R [14]. 369 HCC and 50 normal non-paired samples were used to explore differentially expressed MDGs. The MethylMix algorithm can explore different methylation level and calculate the correlation between gene expression and gene methylation level. We defined MDGs as genes with |logFC| > 0, P < 0.05 and |Cor| > 0.3. Subsequently, consensus nonnegative matrix factorization (CNMF) was applied to conduct consensus clustering based on the integrated gene expression and methylation data of MDGs by the function “ExecuteCNMF” from the R package *CancerSubtypes* [15]. T-SNE based approach was then applied to validate subtype assignments based on mRNA expression data of MDGs. Prediction of previously published HCC molecular classifications [2–4, 18] was performed by conducting nearest template prediction (NTP) analyses (Gene Pattern modules). DEGs among HCC subclasses were identified using *edgeR* package based on raw counts. Genes with an absolute log2 fold change (FC) > 1 (adjusted *P* < 0.01) were defined as DEGs [38].

### Identification of CpG island methylator phenotype

To investigate the relationship between methylation driven genes associated classification and CpG island methylator phenotype, we used previously described approach [7] to identify distinct CpG island methylator phenotype of HCC. In specific, CpGs in the promoter region that have a high standard deviation (SD > 0.2) of methylation level in 369 tumor tissues and low methylation level (mean β value < 0.05) in 50 normal tissues were selected. K-means consensus clustering was performed on these CpGs using the *ConsensusClusterPlus* package in R [39].

### Estimation of metabolism and immune-associated signatures

GSVA is a gene set enrichment method that can estimate the score of certain signatures based on transcriptomic data [40]. Metabolism-relevant (glucose metabolism, amino acid metabolism, lipid metabolism), immune-relevant (antigen presentation MHC class I/II, CD8 T effector, cytolytic activity, IFN), and other HCC progression (ECM, EMT, TGF-β pathway, mismatch repair, DNA damage repair, angiogenesis, cell cycle, differentiation, mTOR pathway, stem, and WNT activation) signatures were achieved from previously published studies [28, 41]. We can quantitatively measure these biological processes by *GSVA* R package. Besides, the absolute abundance of 8 immune cell populations (T cells, CD8^+^ T cells, natural killer cells, cytotoxic lymphocytes, B cell lineage, monocytic lineage cells, myeloid dendritic cells, neutrophils) and 2 nonimmune stromal cell populations (endothelial cells and fibroblasts) was also quantified using MCP-counter algorithm [42].

### Mutation signature and copy number analysis

A predefined set of 30 mutational signatures from the Wellcome Trust Sanger Institute was obtained [43]. Each signature represented a characteristic pattern of 96 possible nucleotide substitution motifs. Relative contribution of each mutational signature for tumor samples was quantified using *deconstructSigs* R package [44], and the parameters were set as following: ‘exome2genome’ trinucleotide-count normalization and signature cutoff at 6%. Prognosis associated mutational signatures (*P* < 0.15) were identified using Cox regression in *survival* package. Copy number variation (CNV) data was downloaded from GDAC Firehose. Then, GISTIC 2.0 (Gene Pattern modules) was used to investigate the significant amplification or deletion events in the regions of the genome [45].

### Development of classification model

The full TCGA dataset (n=369) was randomly split into training and testing cohorts according to the ratio of 4:1, corresponding to 295 and 74 samples. Then, two machine learning (ML) algorithms were used to develop the classification model based on all DEGs. First, RF based variable selection method using OOB error was applied to preliminarily screen for DEGs, and genes accounting for 90% of the cumulative weight were reserved. After primary filtration, a LASSO algorithm, with penalty parameter tuning conducted by k-folds cross-validation (k=20), was used to build the final classification model. Subsequently, the LASSO based classification model was applied to the testing cohort. The predictability of the model was evaluated by confusion matrix and receiver operating characteristic (ROC) curves.

### Statistical analysis

All the computational and statistical analyses were performed using R version 3.6.0 software. The difference between 2 groups was compared using unpaired Student t test (for normally distributed variables) or Mann-Whitney U test (for non-normally distributed variables). For comparisons of 3 or more groups, one-way analysis and Kruskal-Wallis tests were used as parametric and non-parametric methods, respectively. Contingency table variables were analyzed by Chi-square test or Fisher’s exact tests. Survival analysis was carried out using Kaplan Meier methods and was compared by the Log-rank test. A two-tailed p value less than 0.05 was statistically significant.

## Supplementary Material

Supplementary Figures

Supplementary Tables

Supplementary Table 1

Supplementary Table 3

Supplementary Table 7

Supplementary Table 8
