# Supplementary material for: Integrative analysis of DNA methylation and gene expression reveals distinct hepatocellular carcinoma subtypes with therapeutic implications
Source: Aging (Albany NY). 2020 Mar 22;12(6):4970–95. doi: 10.18632/aging.102923 (PMC7138576; doi:10.18632/aging.102923)
Supplement: Supplementary Figures [file aging-12-102923-s006..pdf]

SUPPLEMENTARY FIGURES

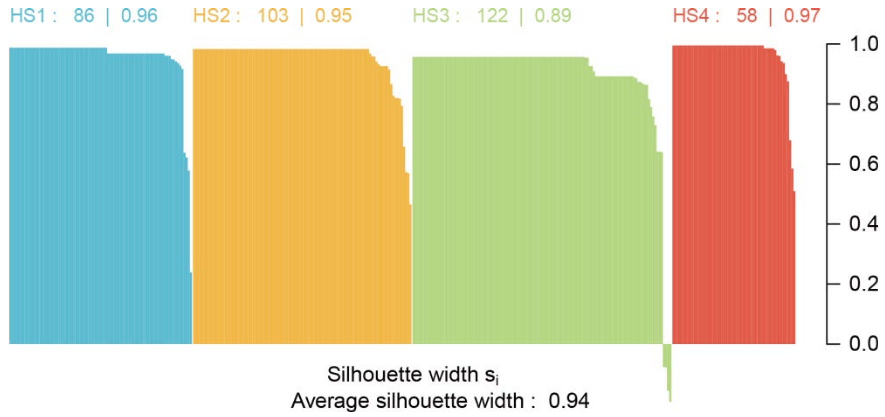

Supplementary Figure 1. Silhouette plot for k = 4 classes.

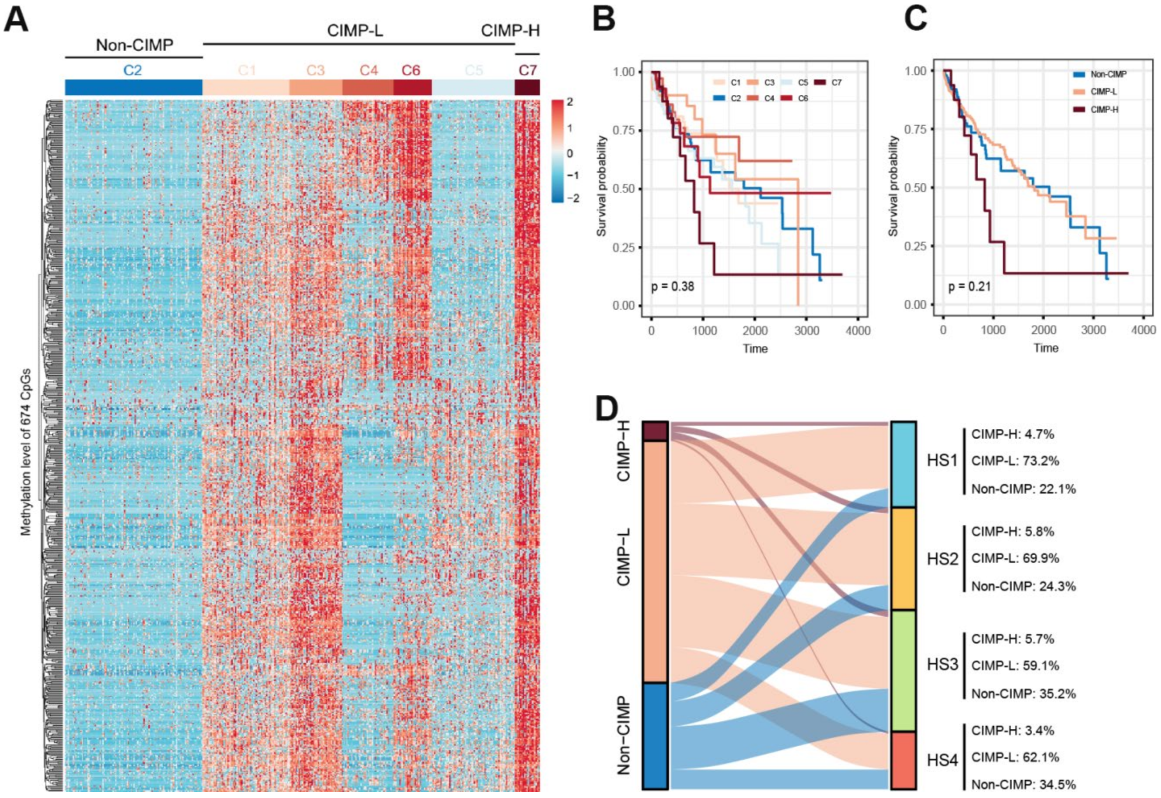

Supplementary Figure 2. Correlation of the HCC subclasses with methylation clusters. (**A**) 7 methylation clusters were obtained using k-means consensus clustering. These clusters were then divided into 3 groups, namely non-CIMP, CIMP-H and CIMP-L. (**B**) Kaplan-Meier survival curves of 7 methylation clusters. Statistical significance of differences was determined by Log-rank test. (**C**) Kaplan-Meier survival curves of 3 methylation groups. (**D**) Sankey plot shows that HS3 and HS4 are associated with non-CIMP group. Statistical significance of differences was determined by Chi-square test. HCC: hepatocellular carcinoma; CIMP: CpG island methylator phenotype.

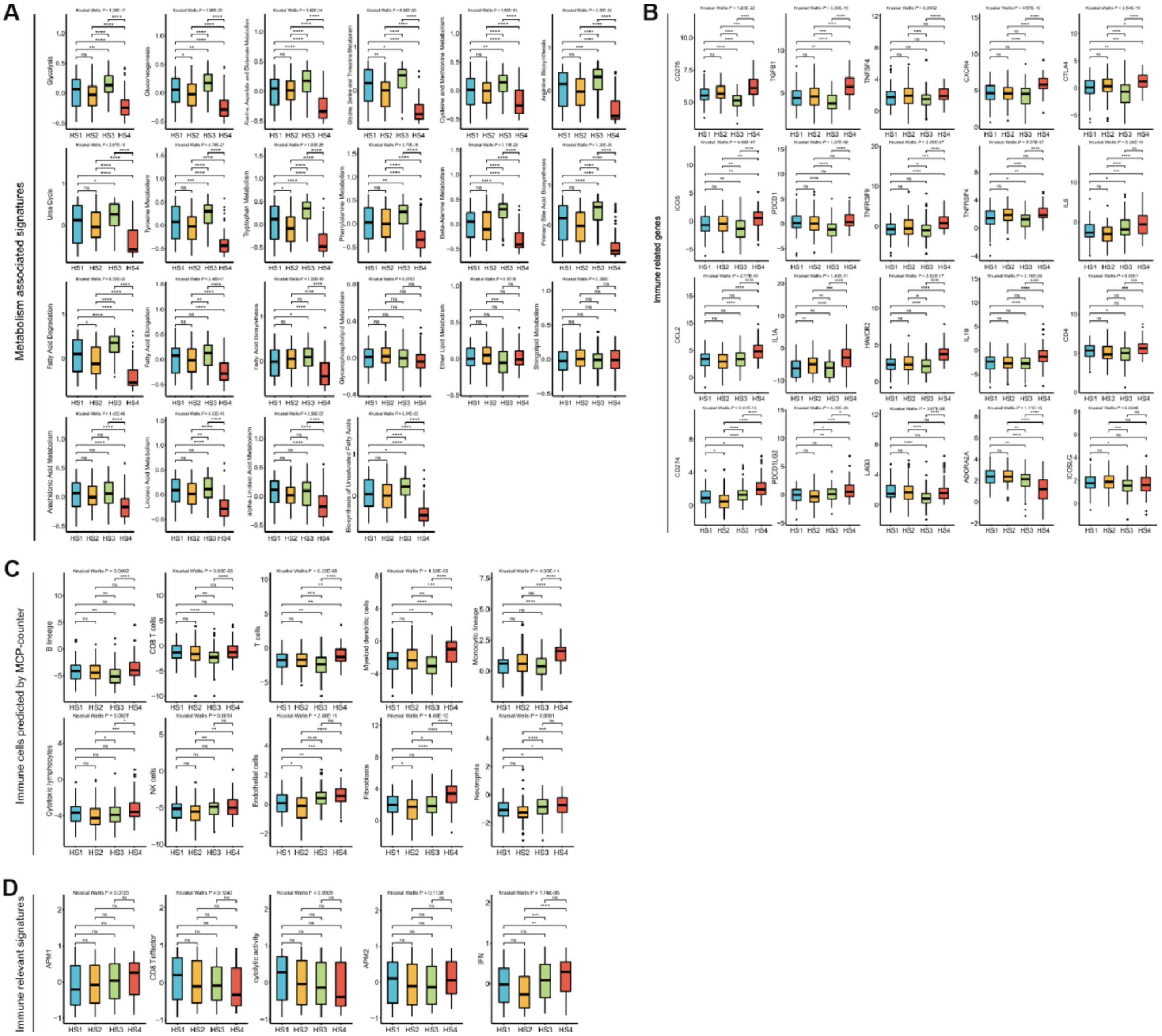

Supplementary Figure 3. Difference of abundance in metabolism and immune-associated signatures among HCC subclasses. (**A**) Box plots (from 25th percentile to the 75th percentile with a line at the median) show the range of abundance in metabolism associated signatures. (**B**) Box plots show the expression level of immune related genes. (**C**) Box plots show the abundance of immune and stromal cell populations. (**D**) Box plots show the abundance of immune-relevant signatures.

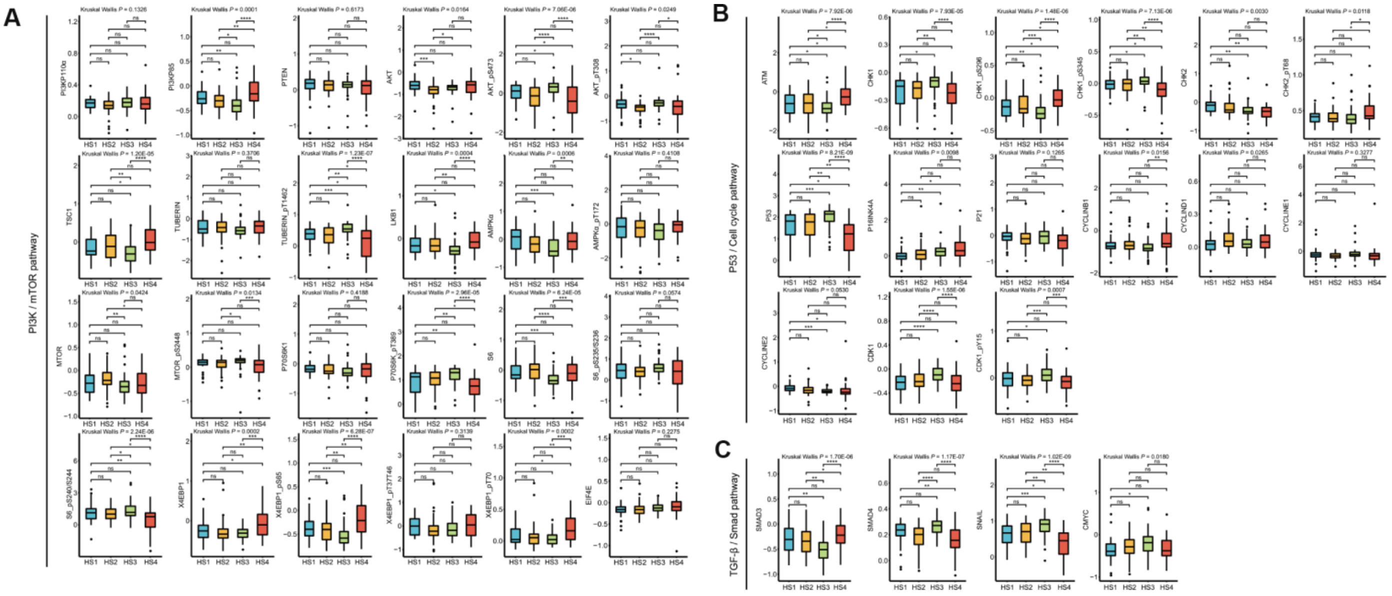

Supplementary Figure 4. Association between HCC classifications and expression level of proteins in 3 pathways. (**A**) Box plots (from 25th percentile to the 75th percentile with a line at the median) show the expression level of proteins in PI3K/mTOR pathway. (**B**) Box plots show the expression level of proteins in P53/Cell cycle pathway. (**C**) Box plots show the expression level of proteins in TGF-β/Smad pathway. HCC: hepatocellular carcinoma.

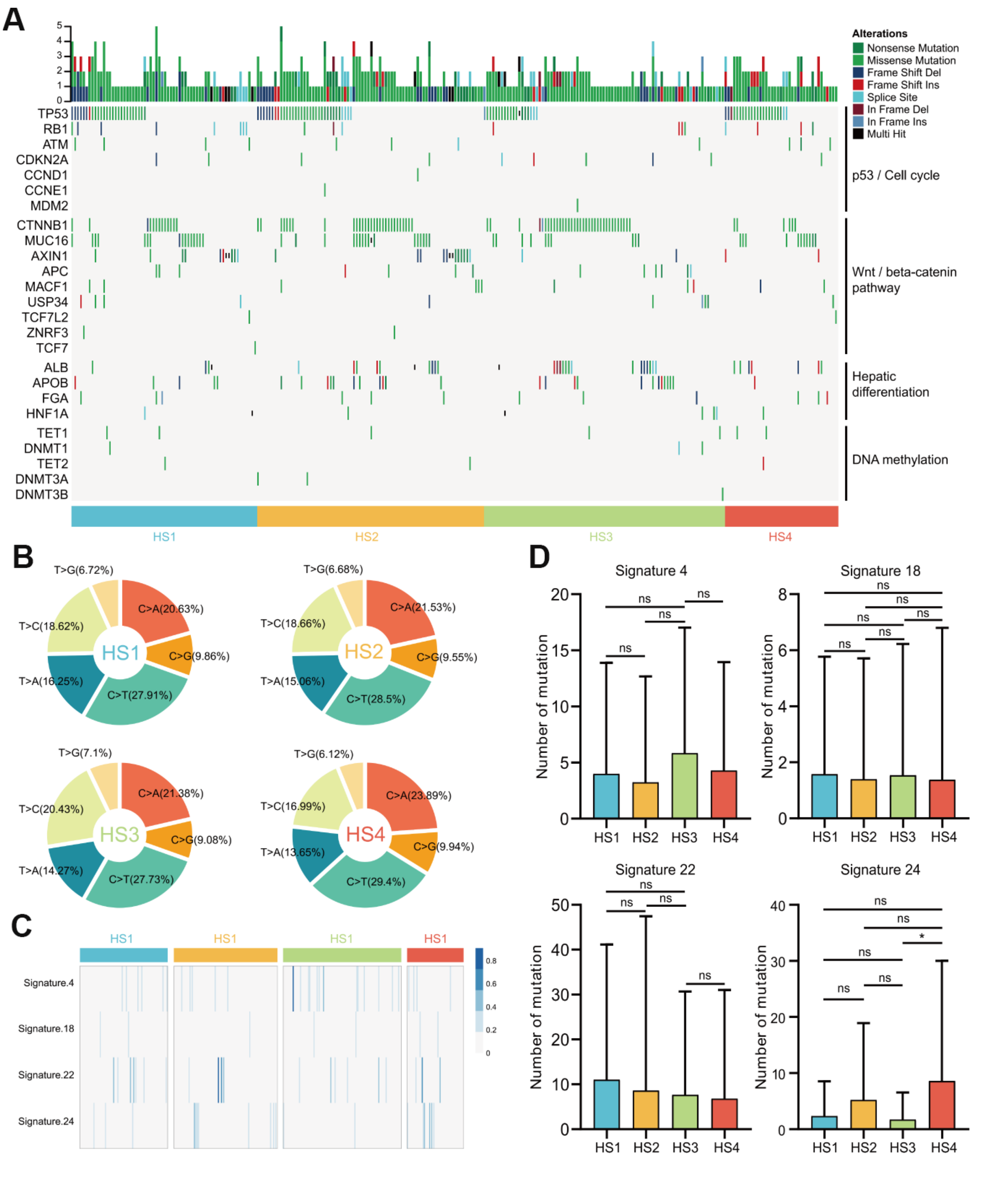

Supplementary Figure 5. Association between HCC classifications and somatic mutation alterations. (**A**) Oncoprint showing mutation status of genes in P53/Cell cycle pathway, Wnt/beta-catenin pathway, hepatic differentiation and DNA methylation (see detailed statistical analysis in Supplementary Table 3). (**B**) Proportion of 6 different single-nucleotide substitutions in HCC classifications are shown. (**C**) Heatmap shows signature weight of 4 prognosis-associated signatures among 4 subclasses. (**D**) Histograms show the difference of mutation number in 4 signatures among HCC subclasses. HCC: hepatocellular carcinoma

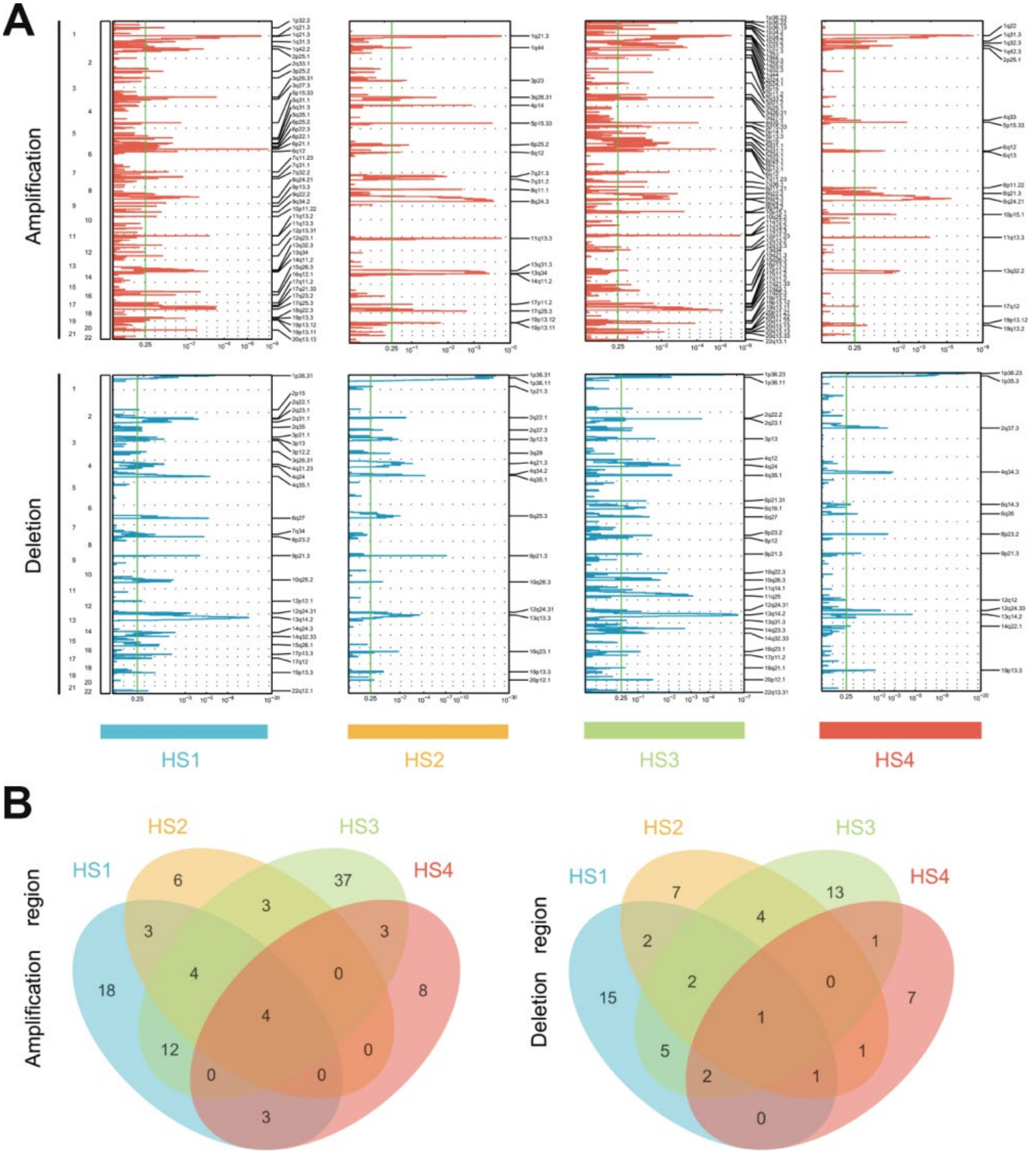

Supplementary Figure 6. Association between HCC classifications and DNA copy number alterations. (**A**) Genome-wide focal amplification (red) and deletion (blue) peaks in 4 HCC subclasses identified by GISTIC2.0. (**B**) Venn diagrams identify the specific/common significant amplification and deletion regions in different HCC subclasses. HCC: hepatocellular carcinoma.
