# Supplementary material for: Integrative analysis of DNA methylation and gene expression reveals distinct hepatocellular carcinoma subtypes with therapeutic implications
Source: Aging (Albany NY). 2020 Mar 22;12(6):4970–95. doi: 10.18632/aging.102923 (PMC7138576; doi:10.18632/aging.102923)
Supplement: Supplementary Tables [file aging-12-102923-s005..pdf]

SUPPLEMENTARY TABLES

Please browse Full Text version to see the data of Supplementary Table 1.

Supplementary Table 1. Results of MethylMix analysis.

Supplementary Table 2. Detailed results from statistical analysis of overall survival and recurrence free survival.

**Results of survival analysis (OS) of distinct HCC classification**					
	**Median survival time**	**95%CI**	**p value (vs HS2)**	**p value (vs HS3)**	**p value (vs HS4)**
HS1	2839	1749-3929	0.0609	0.5308	0.0034
HS2	1622	929-2315	/	0.0786	0.1821
HS3	1818	1213-2423	/	/	0.0048
HS4	1135	450-1820	/	/	/
**Results of survival analysis (RFS) of distinct HCC classification**					
	**Median survival time**	**95%CI**	**p value (vs HS2)**	**p value (vs HS3)**	**p value (vs HS4)**
HS1	1453	806-2100	0.0723	0.6013	0.0911
HS2	828	396-1260	/	0.1228	0.907
HS3	893	587-1199	/	/	0.1196
HS4	489	198-780	/	/	/

Please browse Full Text version to see the data of Supplementary Table 3.

Supplementary Table 3. List of 401 methylation driven genes.

Supplementary Table 4. Clinical Characteristics of TCGA-LIHC cohort.

Clinical Characteristics of TCGA cohort	
**Variable**	**TCGA set (n=369)**
Age	
>55	241
<=55	122
Gender	
female	118
male	245
Viral infection	
HBV	95
HCV	49
HBV and HCV	7
No infection	102
Child-Pugh score	
A	216
B/C	22
Histologic grade	
G1	55
G2	175
G3	116
G4	12
TNM stage	
I/II	254
III/IV	85
AFP level	
Low	181
High	95
Vascular invasion	
None	205
Micro	90
Macro	14
Family history	
No	204
Yes	110

Supplementary Table 5. Detailed results from statistical analysis of mutation characteristics in HCC classifications. HCC: hepatocellular carcinoma.

Mutation characteristics in distinct HCC classification												
	**HS1**	**percentage (%)**	**p-value**	**HS2**	**percentage (%)**	**p-value**	**HS3**	**percentage (%)**	**p-value**	**HS4**	**percentage (%)**	**p-value**
Number of patients	84	100.00		99	100.00		112	100.00		54	100.00	
TP53	26	30.95	ns	33	33.33	ns	19	16.96	0.0009	22	40.74	ns
CTNNB1	13	15.48	0.0402	29	29.29	ns	35	31.25	0.0243	6	11.11	0.0174
MUC16	16	19.05	ns	17	17.17	ns	10	8.93	0.0201	11	20.37	ns
ALB	4	4.76	ns	10	10.10	ns	15	13.39	ns	7	12.96	ns
APOB	5	5.95	ns	11	11.11	ns	12	10.71	ns	1	1.85	ns
AXIN1	11	13.10	0.0495	13	13.13	0.0271	2	1.79	0.0032	2	3.70	ns
RB1	8	9.52	ns	3	3.03	ns	4	3.57	ns	4	7.41	ns
APC	3	3.57	ns	4	4.04	ns	6	5.36	ns	0	0.00	ns
MACF1	2	2.38	ns	4	4.04	ns	3	2.68	ns	2	3.70	ns
FGA	3	3.57	ns	3	3.03	ns	3	2.68	ns	3	5.56	ns
ATM	4	4.76	ns	3	3.03	ns	1	0.89	ns	3	5.56	ns
USP34	4	4.76	ns	1	1.01	ns	4	3.57	ns	1	1.85	ns
CDKN2A	2	2.38	ns	2	2.02	ns	5	4.46	ns	1	1.85	ns
HNF1A	2	2.38	ns	1	1.01	ns	4	3.57	ns	1	1.85	ns
TET1	2	2.38	ns	1	1.01	ns	2	1.79	ns	2	3.70	ns
DNMT1	1	1.19	ns	0	0.00	ns	2	1.79	ns	0	0.00	ns
DNMT3A	0	0.00	ns	2	2.02	ns	0	0.00	ns	0	0.00	ns
TET2	1	1.19	ns	1	1.01	ns	0	0.00	ns	1	1.85	ns
TCF7	1	1.19	ns	0	0.00	ns	0	0.00	ns	0	0.00	ns
TCF7L2	1	1.19	ns	0	0.00	ns	0	0.00	ns	1	1.85	ns
ZNRF3	1	1.19	ns	1	1.01	ns	0	0.00	ns	0	0.00	ns
CCND1	0	0.00	ns	1	1.01	ns	0	0.00	ns	0	0.00	ns
CCNE1	0	0.00	ns	1	1.01	ns	0	0.00	ns	0	0.00	ns
DNMT3B	0	0.00	ns	0	0.00	ns	1	0.89	ns	0	0.00	ns
MDM2	0	0.00	ns	0	0.00	ns	1	0.89	ns	0	0.00	ns

Supplementary Table 6. Results from cox regression of mutation signature.

The results of cox regression of mutation signature		
	**HR**	**p value**
Signature.1	1.011480944	0.638463658
Signature.2	1.002796051	0.947780338
Signature.3	1.000279303	0.959690395
Signature.4	1.012134008	0.13054495
Signature.5	0.993570767	0.225181567
Signature.6	0.959881321	0.373625593
Signature.7	0.973549374	0.514468948
Signature.8	1.002630783	0.734655486
Signature.9	0.986273393	0.25153733
Signature.10	0.943404048	0.409932352
Signature.11	0.975163806	0.524308952
Signature.12	0.986674705	0.277786476
Signature.13	1.016710568	0.649605185
Signature.14	1.034850808	0.471043598
Signature.15	1.040357259	0.190478444
Signature.16	1.000884331	0.616969517
Signature.17	1.052131131	0.228968673
Signature.18	1.030359459	0.096865864
Signature.19	0.99322056	0.764671586
Signature.20	1.03315935	0.279846514
Signature.21	1.010874489	0.555240576
Signature.22	1.005884371	0.020551777
Signature.23	0.986966228	0.754838115
Signature.24	1.019837628	0.0000821
Signature.25	0.992860584	0.616339237
Signature.26	1.002719008	0.810417508
Signature.27	1.01248891	0.669391861
Signature.28	0.985549785	0.443335344
Signature.29	1.004175822	0.825991515
Signature.30	1.019595427	0.177302858

Please browse Full Text version to see the data of Supplementary Tables 7, 8

Supplementary Table 7. Detailed information of specific amplification and deletion regions in HCC classifications. HCC: hepatocellular carcinoma.

Supplementary Table 8. Information of 2835 genes for machine learning.
